# Enhanced Nitrate Reduction Performance of Cu-Doped Nanoporous Co_2_P Electrocatalyst

**DOI:** 10.3390/nano15100753

**Published:** 2025-05-17

**Authors:** Yunduo Huang, Xiechen Zhang, Yanqin Liang, Hui Jiang, Shuilin Wu, Zhaoyang Li, Zhenduo Cui, Shengli Zhu, Zhonghui Gao, Wence Xu

**Affiliations:** 1 School of Materials Science and Engineering, Tianjin University, Tianjin 300350, China; yunduohuang@163.com (Y.H.); zhxch596@163.com (X.Z.); yqliang@tju.edu.cn (Y.L.); h.jiang@tju.edu.cn (H.J.); slwu@pku.edu.cn (S.W.); zyli@tju.edu.cn (Z.L.); zdcui@tju.edu.cn (Z.C.); slzhu@tju.edu.cn (S.Z.); 2 State Key Laboratory of Precious Metal Functional Materials, Tianjin 300350, China; 3 Tianjin Key Laboratory of Composite and Functional Materials, Tianjin 300350, China

**Keywords:** dealloying, nanoporous materials, electrocatalysis, nitrate reduction reaction

## Abstract

Electrocatalytic nitrate reduction to ammonia (NO_3_RR) is a promising approach to recycle nitrogen from nitrate pollutants, yet it remains challenged by low Faradaic efficiency and insufficient NH_3_ production. Herein, Cu-doped nanoporous Co_2_P (np-Co_2−x_Cu_x_P) is reported as electrocatalyst for NO_3_RR, achieving an ammonia yield rate of 30.6 mg h^−1^ cm^−2^ with a Faradaic efficiency of 93.4% at −0.3 V vs. RHE. In-situ spectroscopic analyses indicate that Cu incorporation modifies the surface electronic structure, resulting in the promotion of *H adsorption and *NO_2_^−^ hydrogenation, thereby facilitating efficient ammonia generation.

## 1. Introduction

Ammonia is a widely used industrial raw product and considered an environmentally friendly fuel option for low-carbon energy technologies [1,2,3]. Electrochemical ammonia synthesis offers a promising alternative method for ambient NH_3_ production compared to the traditional energy-intensive Haber-Bosch process [4,5]. Electrochemical nitrate reduction offers a more practical pathway for ammonia synthesis, benefiting from nitrate’s high solubility and the relatively low N=O bond dissociation energy (204 kJ mol^−1^) [6,7,8]. Meanwhile, nitrate, commonly found in wastewater due to human activities, presents an appealing opportunity for converting pollutants into valuable ammonia from an environmental standpoint [9,10,11,12]. This highlights the importance of developing efficient electrocatalysts that can facilitate the multi-electron transfer process and enhance NH_3_ selectivity under ambient conditions. However, the NO_3_^−^ conversion process is generally regarded as the rate-determining step, which has a low probability of occurrence due to ubiquitous energy-scaling relations [13,14]. Consequently, high overpotentials are required to overcome sluggish kinetics, which may trigger the competing hydrogen evolution reaction (HER), which undesirably consumes electrons and reduces nitrate reduction efficiency, thereby, reducing NH_3_ selectivity. Therefore, the development of catalysts that accelerate nitrate activation while suppressing HER at low overpotentials is critical for improving selectivity and energy efficiency.

Recent studies have witnessed breakthroughs in transition metal-based NO_3_RR catalysts, along with strategies aimed at enhancing activity and selectivity [15,16]. Among these, cobalt phosphides are highly effective for generating active hydrogen species and demonstrate exceptional catalytic performance towards NO_3_RR [17,18,19]. The cobalt centers with partial positive charges can effectively adsorb nitrate anions, while phosphorus centers with partial negative charges can serve as proton-acceptor sites. Tailoring the local electronic structure of cobalt phosphides by doping with other metals such as copper is a promising approach to optimize the adsorption strength of reaction intermediates and modulate hydrogen adsorption behavior. Unlike heterostructure engineering, metal doping can maintain the phase integrity of the host material while enabling fine-tuning of its surface reactivity. For instance, Cu-doped Fe_3_O_4_ catalysts have demonstrated nearly 97% Faradaic efficiency and high ammonia yield in NO_3_RR, attributed to the favorable electronic modulation introduced by Cu doping [20]. Similarly, Co-doped Fe@Fe_2_O_3_ catalysts derived from metal–organic frameworks exhibit enhanced NO_3_RR performance, where Co doping adjusts the Fe d-orbitals, improving intermediate adsorption and suppressing competing hydrogen evolution reactions [21]. In the context of cobalt phosphide systems, doping with transition metals like Fe, Ni has been explored to modulate electronic properties and catalytic activity. Studies indicate that such doping can influence the crystal structure, morphology, and electronic characteristics of Co_2_P-based catalysts, thereby affecting their performance in reactions [22,23]. However, conventional phosphide synthesis methods that often require toxic reagents or high-temperature treatments [24]. In addition, studies on Cu-doped Co_2_P for nitrate reduction remain limited, the appropriate modification of electronic structure by Cu doping for the metal phosphides deserves further investigation.

Herein, we report green and facile approach to prepare Cu-doped nanoporous Co_2_P (np-Co_2−x_Cu_x_P) via a simple dealloying method for efficient electrochemical nitrate reduction. The catalyst achieves a notable ammonia production rate of 30.6 mg h^−1^ cm^−2^ and maintains a high Faradaic efficiency of 93.4% at −0.3 V versus RHE. Notably, it delivers a high current density exceeding 600 mA cm^−2^ at −0.4 V versus RHE, indicating excellent catalytic kinetics and conductivity. X-ray photoelectron spectroscopy (XPS) indicates that Cu incorporation modifies the electronic environments of Co and P atoms, which in turn facilitates the adsorption and transformation of crucial reaction intermediates. In-situ attenuated total reflection surface-enhanced infrared absorption spectroscopy (ATR-SEIRAS) results indicate a reduced onset potential for intermediate formation, which facilitates *H adsorption and *NO_2_ hydrogenation. These results demonstrate that the np-Co_2−x_Cu_x_P catalyst, prepared through a facile and scalable method, offers excellent activity and selectivity toward ammonia production. By tuning the Cu content, the electronic structure and catalytic behavior of cobalt phosphide can be effectively modulated, showing great promise for practical nitrate-to-ammonia conversion applications.

## 2. Materials and Methods

### 2.1. Materials Fabrication

The precursor alloys were synthesized using a vacuum high-frequency induction melting furnace. Co, Cu, and Co_2_P were weighed according to atomic ratios of Co:Cu:P = 80:0:20, 75:5:20, 70:10:20, and 65:15:20, respectively, and then melted to form the desired alloys (Figure S1). The corresponding mass of each component is listed in Table S1. According to the actual Cu ratios, the four samples with Co:Cu:P = 80:0:20, 75:5:20, 70:10:20, and 65:15:20 were named as np-Co_2_P, np-Co_1.94_Cu_0.06_P, np-Co_1.34_Cu_0.66_P, and np-Co_1.04_Cu_0.96_P, respectively. The resulting melt was then remelted in a quartz cone tube with one end opening and quickly ejected onto a copper wheel rotating at 3500 rpm. This rapid cooling process produced the precursor alloy ribbons. The dealloying process was performed electrochemically in 1 M HCl at −0.05 V, employing a standard three-electrode setup [25]. Following dealloying, the resulting ribbons were thoroughly washed in deionized water and ethanol.

### 2.2. Materials Characterization

The phase composition was analyzed using X-ray diffraction (XRD model: Bruker D8 GmbH, Karlsruhe, Germany) with Cu Kα radiation. Morphological, microstructural, and compositional characterizations were carried out using a transmission electron microscope (TEM: JEOL JEM-2100F Akishima, Tokyo, Japan) coupled with an energy-dispersive X-ray spectrometer (EDS) and a scanning electron microscope (SEM: S-4800 Hitachi Tokyo, Japan). The elemental valence states were determined by X-ray photoelectron spectroscopy (XPS; Axis Supra Kratos Analytical Ltd., Manchester, UK).

### 2.3. Electrochemical Measurements

The electrocatalytic reactions were performed in an H-type electrochemical cell, with the anode and cathode compartments separated by a Nafion membrane. Before assembly, the Nafion membrane was handled with a 5 wt.% hydrogen peroxide solution at 80 °C for 60 min, followed by rinsing with deionized water for 30 min. It was then boiled in a 5 wt.% sulfuric acid solution at 80 °C for 60 min and washed again in deionized water for 30 min.

In the cathode compartment, the np-Co_2_P or np-Co_2−x_Cu_x_P catalyst was applied as the working electrode. A platinum mesh served as the counter electrode in the anode compartment, and an Ag/AgCl electrode was used as the reference electrode. All electrochemical tests were conducted at room temperature. The potentials were converted to the reversible hydrogen electrode (RHE) using the following Equation (1):

(1) $$E_{RHE}=E_{Ag/AgCl}+0.0591\times pH+0.198$$

### 2.4. Determination of NH_3_

Ammonia (NH_3_) levels in the electrolyte were determined by the indophenol blue method. Extract the reaction solution from the reaction chamber and dilute it to 2 mL at a specific ratio. Then, 1 M NaOH solution with salicylic acid and sodium citrate, 0.05 M NaClO and C_5_FeN_6_Na_2_O (1 wt.%) was added to the diluted 2 mL of electrolyte. After two hours, the absorbance at 655 nm was measured using a UV fluorescence photometer. For comparison, a series of standard absorbance curves at different concentrations were prepared using NH_4_Cl. The ammonia production rate and Faraday efficiency were determined using the following equations:

The NH_3_ yield was calculated using Equation (2):

(2) $${Yield}_{\;{\mathrm{NH}}_{3}}=\frac{C_{{\mathrm{NH}}_{3}}•V}{t•S}$$

where *C*_NH3_ refers to the NH_3_ concentration, which was measured using a UV-Vis spectrophotometer (UV-2700 Shimadzu Corporation, Kyoto, Japan). and calculated based on the standard curve. *V* denotes the electrolyte volume, *t* corresponds to the electrolysis duration, and *S* represents the surface area of the electrode.

Faradaic efficiency (FE) was calculated using Equation (3):

(3) $$\mathrm{FE}=\frac{8F•C_{{\mathrm{NH}}_{3}}•V}{Q}$$

where 8 represents the total electrons transferred during the reduction of nitrate to ammonia, *F* refers to the Faraday constant (96,485 C/mol), *C*_NH3_ denotes the concentration of NH_3_, *V* indicates the total volume of electrolyte used in the reaction, and *Q* represents the actual charge used during the test. The corresponding ammonia standard curves for different concentrations are shown in Figure S2.

### 2.5. In-Situ ATR-SEIRAS Measurements

In-situ ATR-SEIRAS was conducted using a Thermo Scientific IS50 spectrometer (Thermo Fisher Scientific Inc., Waltham, MA, USA). The standard three-electrode system was employed, with a platinum mesh as the counter electrode and an Ag/AgCl electrode as the reference electrode. The working electrode was prepared by sonicating the catalyst to form an ink, which was drop-cast onto a gold-sputtered silicon column. A Polaris long-life air-cooled mid/far-infrared source was used as the infrared light source. Infrared spectra were collected at room temperature and atmospheric pressure, with a constant applied potential. The spectra were acquired over a collection time of 90 s.

## 3. Results and Discussion

**Figure 1:**
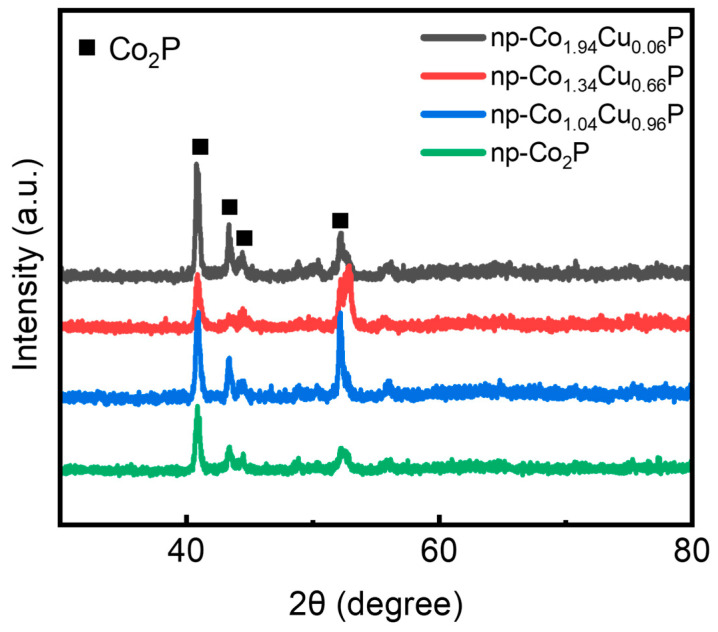
XRD pattern of dealloyed alloys with different Cu contents.

The np-Co_2−x_Cu_x_P catalyst was prepared by selectively etching the Co phase from the Co-Cu-P precursor alloy (Figure S1). The Co-Cu-P precursor alloys were prepared by melt-spinning method. The precursor alloys with designed compositions were melted into liquid state in quartz tubes and then ejected on a rotating copper roller to realize rapid cooling. During the solidification process, the Cu atoms trends to alloy with metallic Co and dope into the Co_2_P in the nucleation and growth process, forming the metallic Co phase, Co_0.52_Cu_0.48_ alloy phase and Co_2_P phase in the precursor alloy. The phase compositions of these precursor alloys were examined by X-ray diffraction (XRD). As shown in Figure S3, the diffraction peaks located at 44.4°, 51.5°, and 75.9° are attributed to metallic Co, while peaks at 41.1°, 43.2°, and 52.3° correspond to the Co_2_P phase [25,26,27]. With increasing Cu content, additional peaks at 50.6° and 74.3° emerge, which can be assigned to the Co_0.52_Cu_0.48_ alloy phase [28,29]. This indicates that Cu atoms gradually occupy the lattice sites of Co, forming a solid solution alloy phase when the Cu content becomes sufficiently high. Compared with the binary Co_80_P_20_ precursor, the Cu-doped samples show no significant phase difference at low Cu contents; however, a noticeable increase in the intensity of Co_0.52_Cu_0.48_-related peaks is observed at higher Cu loadings, suggesting enhanced alloying behavior for Cu. After electrochemical dealloying, the Co and Co_0.52_Cu_0.48_ alloy phases are selectively dissolved, forming a porous Co_2_P structure (Figure 1). The complete disappearance of metallic Co and Co_0.52_Cu_0.48_ peaks confirms their full removal during dealloying. This process is critical for generating high surface area and accessible active sites [25]. Figure S4 shows the enlarged XRD patterns focusing on the (201) plane of Co_2_P for all samples. A clear shift of the diffraction peak towards lower angles with increasing Cu content is observed, indicating a lattice expansion due to the incorporation of Cu atoms, which possess a larger atomic radius than Co. This confirms the successful substitutional doping of Cu into the Co_2_P lattice, leading to the modulation of its crystal structure. Therefore, Co not only facilitates the formation of a dealloyable structure but also indirectly influences the final composition and morphology of the catalyst.

**Figure 2:**
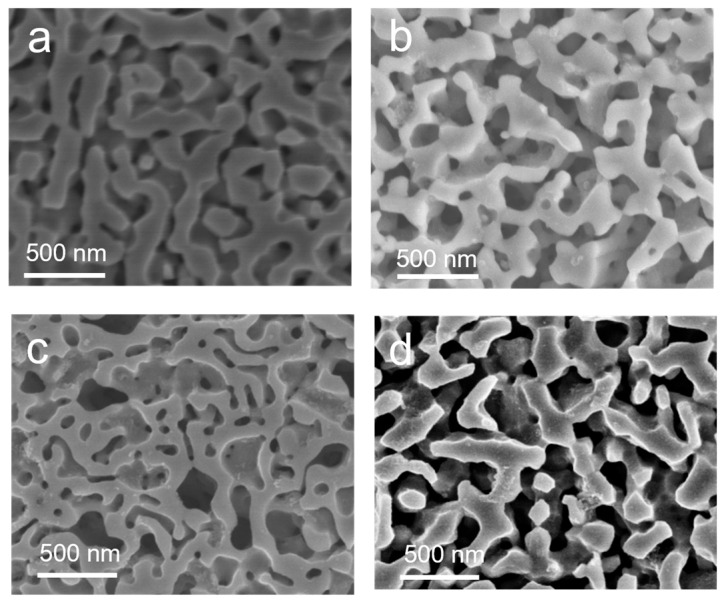
SEM image of (**a**) np-Co_2_P (**b**) np-Co_1.94_Cu_0.06_P (**c**) np-Co_1.34_Cu_0.66_P (**d**) np-Co_104_Cu_0.96_P alloy.

To further investigate the influence of Cu content on the morphology and composition of np-Co_2−x_Cu_x_P, SEM and EDX analyses were performed. EDX results (Table S2) confirm that the Cu content can be effectively tuned from 1.8% to 24.05% and the corresponding ICP analysis (Table S3) further verifies the actual elemental composition is consistent with the designed values. As shown in Figure 2, all samples exhibit interconnected nanoporous structures, but with distinct features depending on Cu content. The np-Co_2_P sample displays uniform ~200 nm pores, resulting from selective removal of the Co phase and retention of the Co_2_P framework. With increased Cu content, smaller pores (~50 nm) emerge alongside the larger ones due to the involvement of Co_0.52_Cu_0.48_ alloy in the dealloying process, which enhances surface area and active site density. However, further increasing Cu content leads to coarsened porous structures (~300 nm), likely due to excessive corrosion of Co–Cu phases, resulting in reduced surface area.

**Figure 3:**
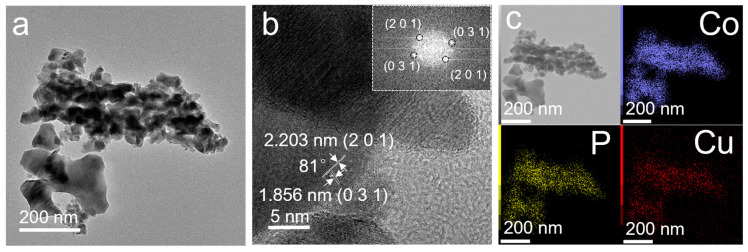
TEM image (**a**), HRTEM and FFT patterns for HRTEM images (**b**), and EDS elemental mapping (**c**) of dealloyed np-Co_1.34_Cu_0.66_P.

To gain deeper insights into the morphology, phase composition, and elemental distribution of the np-Co_1.34_Cu_0.66_P, transmission electron microscopy (TEM) and energy-dispersive X-ray spectroscopy (EDS) analyses were conducted. As shown in Figure 3a, the TEM image reveals a hierarchical porous structure, consistent with the SEM observations. The high-resolution TEM (HRTEM) image and FFT patterns in Figure 3b displays well-defined lattice fringes with interplanar spacings of 2.203 Å and 1.856 Å, corresponding to the (201) and (031) planes of Co_2_P, respectively. The FFT pattern shows two pairs of centrosymmetric diffraction spots, corresponding to the (201) and (031) planes of Co_2_P, which further confirms the good crystallinity and specific orientation of the sample [30,31]. The measured angle between these two planes is approximately 81°, which aligns well with theoretical values, further confirming the Co_2_P phase of the material. Elemental mapping via EDS (Figure 3c) shows a homogeneous distribution of Co, P, and Cu throughout the porous framework, indicating that Cu atoms are uniformly doped into the Co_2_P lattice.

**Figure 4:**
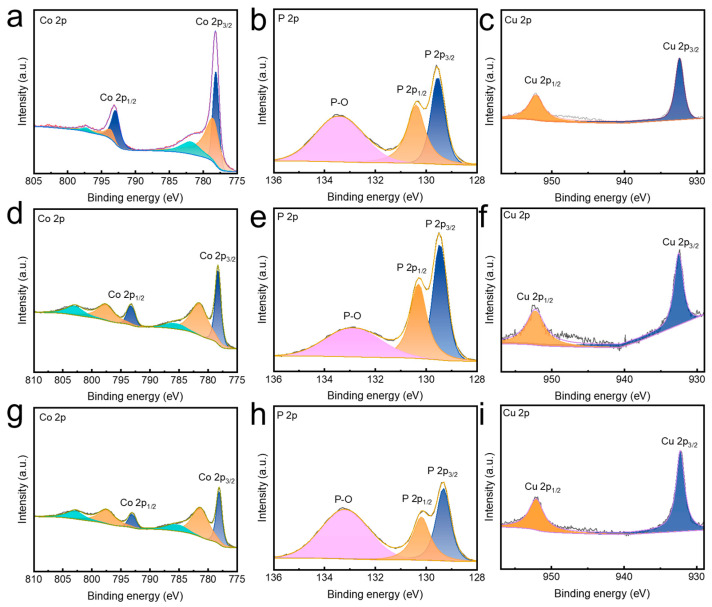
XPS spectra of (**a**–**c**) Co 2p, P 2p, and Cu 2p for np-Co_1.94_Cu_0.06_P, (**d**–**f**) Co 2p, P 2p, and Cu 2p for np-Co_1.34_Cu_0.66_P, and (**g**–**i**) Co 2p, P 2p, and Cu 2p for np-Co_1.04_Cu_0.96_P.

To gain insight into the electronic structure modulation induced by Cu incorporation, X-ray photoelectron spectroscopy (XPS) was conducted on the np-Co_2−x_Cu_x_P samples, as displayed in Figure 3. The Co 2p spectra (Figure 4a,d,g) show two main peaks around 778.1 eV and 793.1 eV, corresponding to Co 2p^3/2^ and Co 2p^1/2^ of Co^0^. Additional peaks at 781.1 eV and 797.5 eV are attributed to Co^2+^ and Co^3+^ species, likely due to surface oxidation during measurement [18,32]. In the P 2p spectra (Figure 4b,e,h), the peaks at 129.3 eV and 130.2 eV are assigned to the 2p^3/2^ and 2p^1/2^ of metal phosphides, while the peak at 133.2 eV is associated with oxidized phosphorus [33,34]. For Cu (Figure 4c,f,i), the characteristic Cu-P peak appears at 932.2 eV (Cu 2p^3/2^) and 952.1 eV (Cu 2p^1/2^), confirming the successful incorporation of Cu. As the Cu content increases, a positive shift of Co 2p and a negative shift of P 2p binding energies are observed, indicating electron redistribution due to Cu doping-where Cu lowers the electron density around Co and promotes electron transfer from Co to P. Notably, the Cu 2p^3/2^ peak of np-Co_1.34_Cu_0.66_P appears at the highest binding energy (932.5 eV), suggesting the strongest interaction between Cu and the Co_2_P matrix, which is beneficial for enhancing electron transfer during electrocatalytic nitrate reduction [35,36].

**Figure 5:**
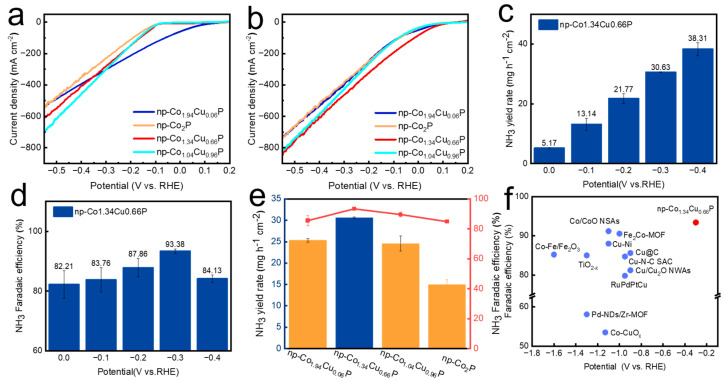
(**a**) LSV curves of np-Co_2−x_Cu_x_P catalysts in 1 M KOH; (**b**) LSV curves in 1 M KOH + 0.1 M NaNO_3_; (**c**) NH_3_ yield and (**d**) Faradaic efficiency of np-Co_1.34_Cu_0.66_P at different applied potentials; (**e**) NH_3_ yield and Faradaic efficiency of np-Co_2−x_Cu_x_P at −0.3 V; (**f**) Comparison of Faradaic efficiency with recently reported high-performance NO_3_RR catalysts.

To further evaluate the electrocatalytic nitrate reduction performance of np-Co_2−x_Cu_x_P catalysts, a series of electrochemical measurements were conducted. As shown in Figure 5a,b, np-Co_1.34_Cu_0.66_P exhibits the highest NO_3_RR current density among all tested samples, especially at low overpotentials, significantly outperforming np-Co_2_P. This indicates that appropriate Cu doping effectively enhances the catalytic activity of Co_2_P. The catalytic activity was further evaluated by the potentiostatic method. The corresponding i–t curve is shown in Figure S5. Figure 5c,d display the NH_3_ yield and Faradaic efficiency (FE) of np-Co_1.34_Cu_0.66_P under various potentials. As the potential becomes more negative, NH_3_ yield increases, while FE peaks at −0.3 V with a maximum of 93.7%, which can be attributed to the competitive hydrogen evolution at high overpotential. Figure 5e demonstrates the NH_3_ yield and FE of different samples at −0.3 V. The np-Co_1.34_Cu_0.66_P catalyst achieves the highest NH_3_ yield and FE among the Co_2_P-based catalysts. To assess the long-term durability, a 10-h electrolysis test was conducted at −0.3 V, during which both NH_3_ yield and FE remained stable, indicating excellent catalytic stability (Figure S6). In addition, no obvious difference can be detected before and after stability test, confirming the high stability of the nanoporous Cu doped Co_2_P. To verify that the detected ammonia originates from the electrochemical reaction, control experiments without applied potential and without nitrate were conducted. In both cases, no significant ammonia was detected, as shown in Figure S7. To further confirm the suppression of the hydrogen evolution reaction by Cu incorporation, gas chromatography (GC) measurements were conducted. As shown in Figure S8, the np-Co_1.34_Cu_0.66_P exhibited a H_2_ Faradaic efficiency of 2.59% at −0.3 V vs. RHE, which is lower than that of np-Co_2_P (6.08%), indicating the suppression of HER by Cu doping. This trend can be attributed to the fact that pure Co_2_P has limited water dissociation ability, resulting in insufficient *H for NO_3_^-^ hydrogenation at low potentials. Moderate Cu doping promotes H_2_O dissociation and provides more reactive hydrogen, improving NH_3_ selectivity and FE. However, excessive Cu content leads to weakened NO_3_^−^ and intermediate adsorption. A comparison of np-Co_1.34_Cu_0.66_P with other state-of-the-art catalysts is presented in Figure 5f and Table S4, indicating that the np-Co_1.34_Cu_0.66_P exhibits superior catalytic performance compared with other NO_3_RR electrocatalysts [21,37,38,39,40,41,42,43,44,45,46].

**Figure 6:**
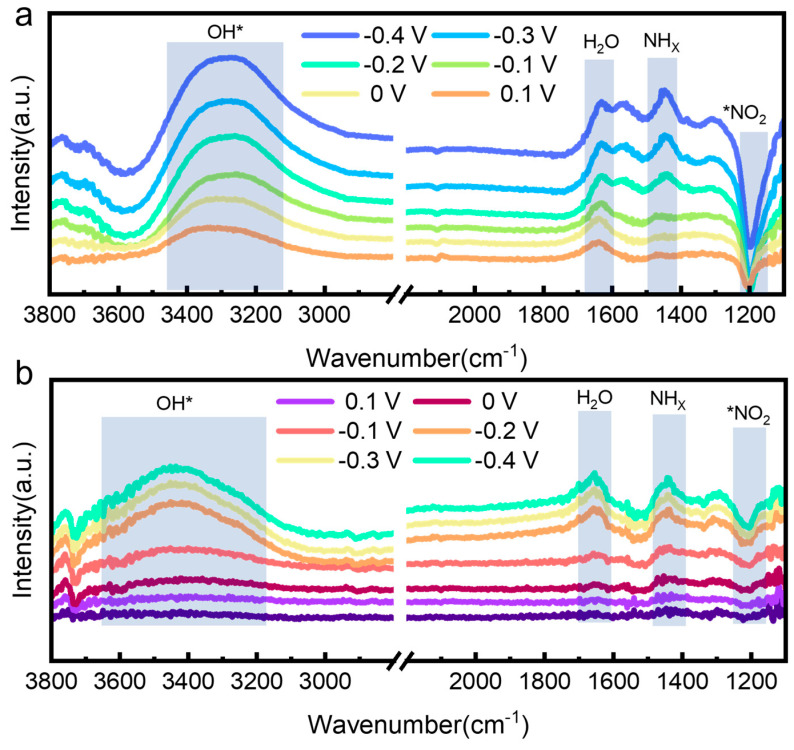
In-situ ATR-SEIRAS spectra of (**a**) np-Co_2_P and (**b**) np-Co_1.34_Cu_0.66_P.

To further explore the enhanced mechanism caused by Cu doping, in situ ATR-SEIRAS was conducted to monitor reaction intermediates. As shown in Figure 6, the spectra of np-Co_2_P and np-Co_1.34_Cu_0.66_P display characteristic peaks at 1209, 1438, and 1632 cm^−1^, corresponding to *NO_2_, *NH_x_, and H_2_O species, respectively [11,47,48,49]. For np-Co_2_P, the *NH_x_ signal emerges at −0.2 V and strengthens as the potential becomes more negative, indicating progressive reduction of nitrate. The presence of *NO_2_ at 0.2 V implies that Co_2_P enables nitrate activation at low overpotential, but insufficient hydrogen availability results in incomplete reduction and byproduct formation. In contrast, the *NH_x_ signal for np-Co_1.34_Cu_0.66_P appears at 0 V, suggesting improved conversion of *NO_2_ to *NH_x_ due to Cu incorporation [50,51]. Moreover, a blue shift in the O-H stretching vibration (from 3300 to 3400 cm^−1^) indicates enhanced water dissociation on the Cu-doped surface, leading to more active hydrogen species that facilitate nitrate hydrogenation and promote catalytic activity [52,53,54].

## 4. Conclusions

In summary, nanoporous Cu-doped Co_2_P (np-Co_2−x_Cu_x_P) electrocatalysts were successfully fabricated via chemical dealloying method and used as the electrocatalyst for nitrate reduction reaction to ammonia. Structural characterizations demonstrate that moderate Cu incorporation not only retained the nanoporous framework but also modulated the electronic structure of Co_2_P. At −0.3 V vs. RHE, np-Co_1.34_Cu_0.66_P delivers an impressive ammonia production rate of 30.6 mg h^−1^ cm^−2^, along with a notable Faradaic efficiency of 93.4%, outperforming both pristine Co_2_P and other Cu-doped variants. In-situ ATR-SEIRAS experiments reveal that Cu doping facilitates water dissociation and provides more active hydrogen species for efficient nitrate hydrogenation, thereby promoting the transformation of *NO_2_ to *NH_x_ intermediates. This study highlights the crucial role of electronic structure engineering in optimizing NO_3_RR performance and offers a promising strategy for designing efficient non-noble metal catalysts for sustainable ammonia synthesis.

## Data Availability

The original contributions presented in this study are included in the article/Supplementary Materials. Further inquiries can be directed to the corresponding author.

## Supplementary Materials

The following supporting information can be downloaded at: https://www.mdpi.com/article/10.3390/nano15100753/s1.
