# Supplementary material for: Enhanced Nitrate Reduction Performance of Cu-Doped Nanoporous Co2P Electrocatalyst
Source: Nanomaterials (Basel). 2025 May 17;15(10):753. doi: 10.3390/nano15100753 (PMC12114531; doi:10.3390/nano15100753)
Supplement: Supplementary file 1 [file nanomaterials-15-00753-s001.zip › nanomaterials-3627726-supplementary.pdf]

Enhanced Nitrate Reduction Performance of Cu-Doped Nanoporous Co_2P Electrocatalyst

Yunduo Huang [1], Xiechen Zhang [1], Yanqin Liang [1,2,3], Hui Jiang [1,2,3], Shuilin Wu [1,2], Zhaoyang Li [1,2,3], Zhenduo Cui [1,2], Shengli Zhu [1,2,3], Zhonghui Gao [1,2,*] and Wence Xu [1,2,*]

1 School of Materials Science and Engineering, Tianjin University, Tianjin 300350, China
2 State Key Laboratory of Precious Metal Functional Materials, Tianjin 300350, China
3 Tianjin Key Laboratory of Composite and Functional Materials, Tianjin 300350, China
* Correspondence: zhgao@tju.edu.cn (Z.G.); wcxu@tju.edu.cn (W.X.)

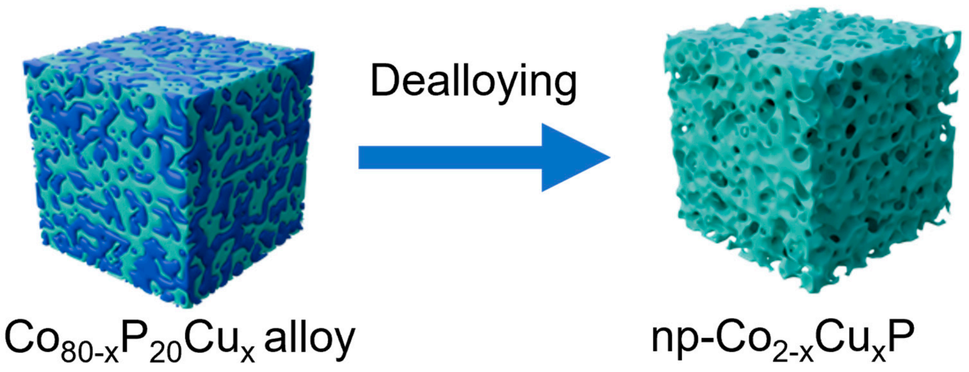

Figure. S1. Sample preparation diagram

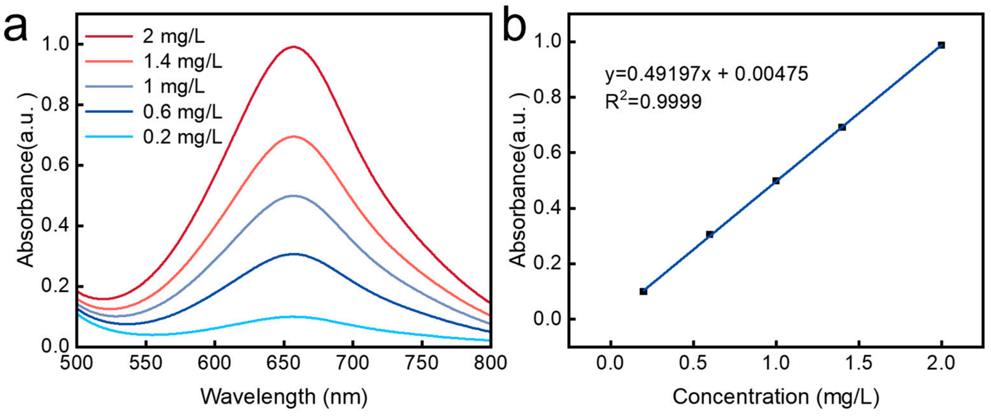

Figure. S2. (a) UV-Vis absorption curves of standard samples and (b) corresponding NH_4^+ calibration curve.

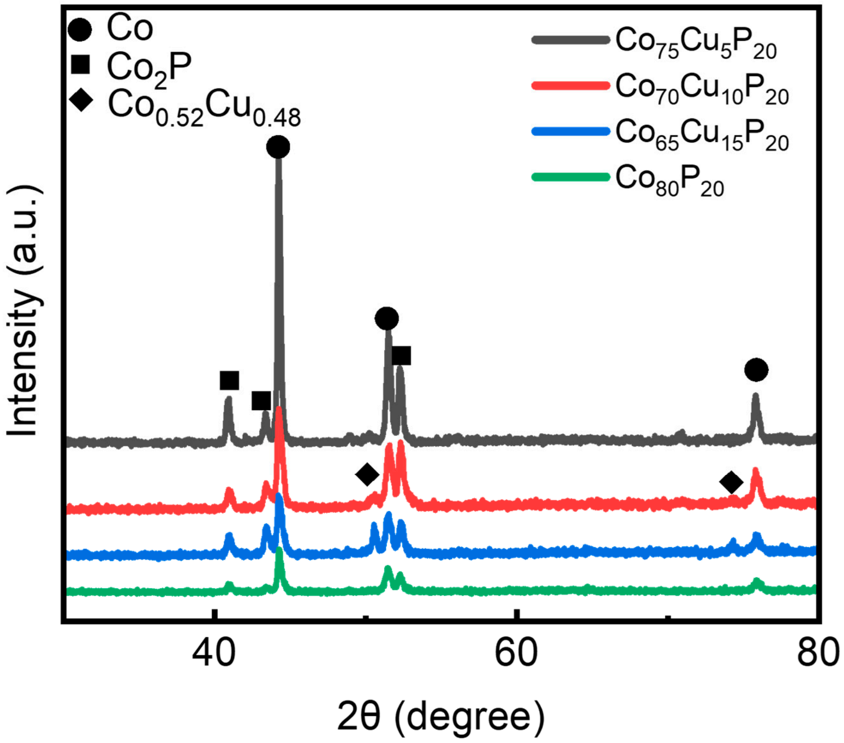

Figure. S3. XRD patterns of alloys with different Cu contents

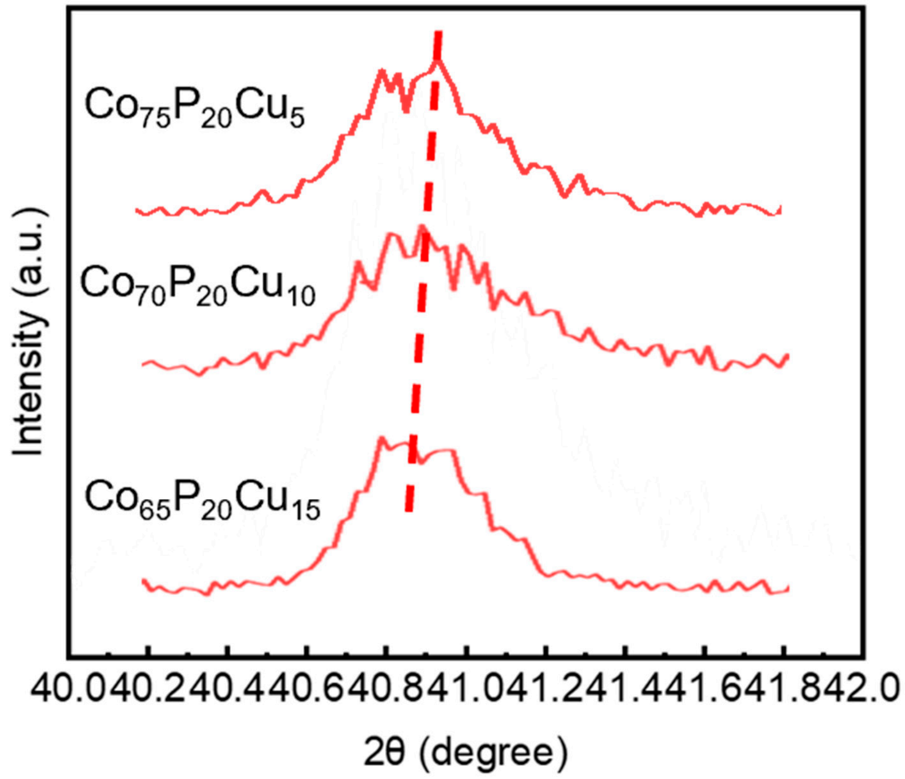

Figure. S4. XRD patterns focusing on the (201) plane of Co_2P

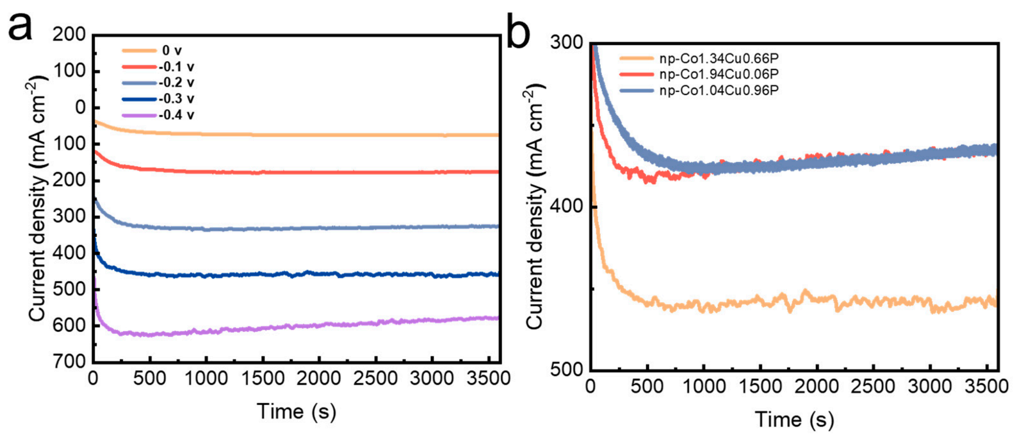

Figure. S5. (a) Time-current curve of np-$Co_{1.34}Cu_{0.66}P$ at different potentials during electrochemical nitrate reduction. (b) Time-current curve of np-$Co_{2-x}Cu_xP$ at -0.3 V

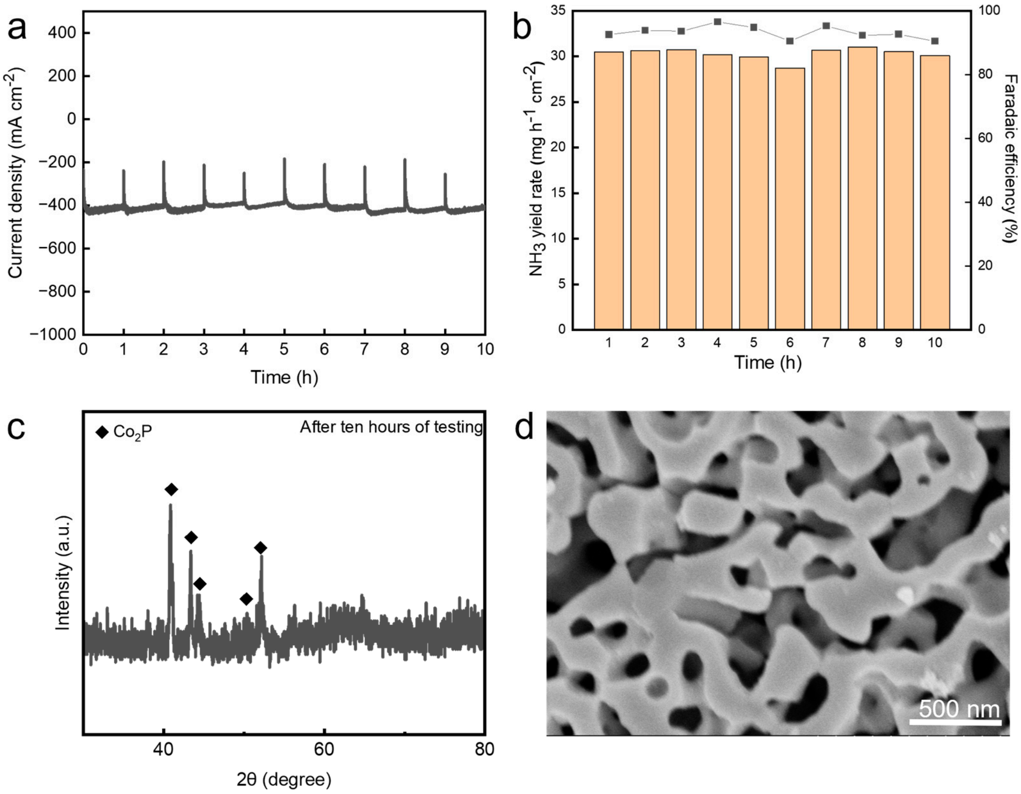

Figure. S6. (a) i-t curves of np-$Co_{1.34}Cu_{0.66}P$ for 10 h electrolysis; (b) Corresponding Faradaic efficiency and NH_3 yield of np-$Co_{1.34}Cu_{0.66}P$ over 10 h; (c) XRD pattern of np-$Co_{1.34}Cu_{0.66}P$ after stability test; (d) SEM image of np-$Co_{1.34}Cu_{0.66}P$ after 10 h electrolysis.

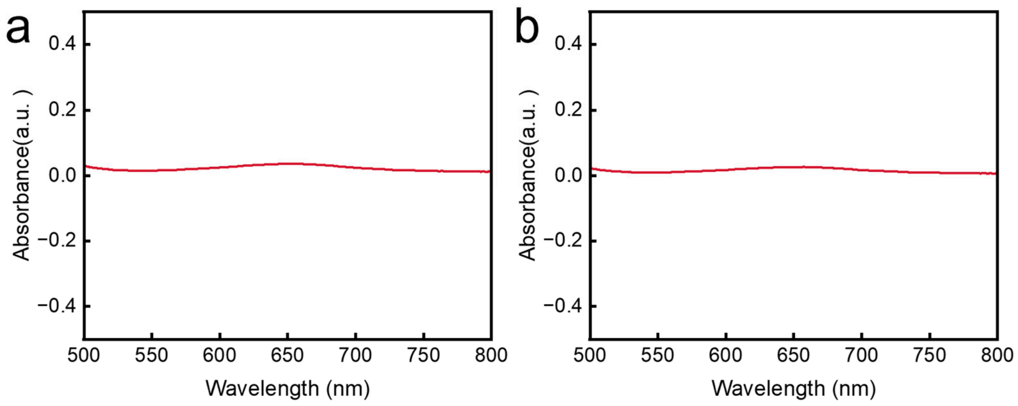

Figure. S7. (a) UV-Vis absorption curves without applied potential; (b) UV–Vis absorption curves without nitrate.

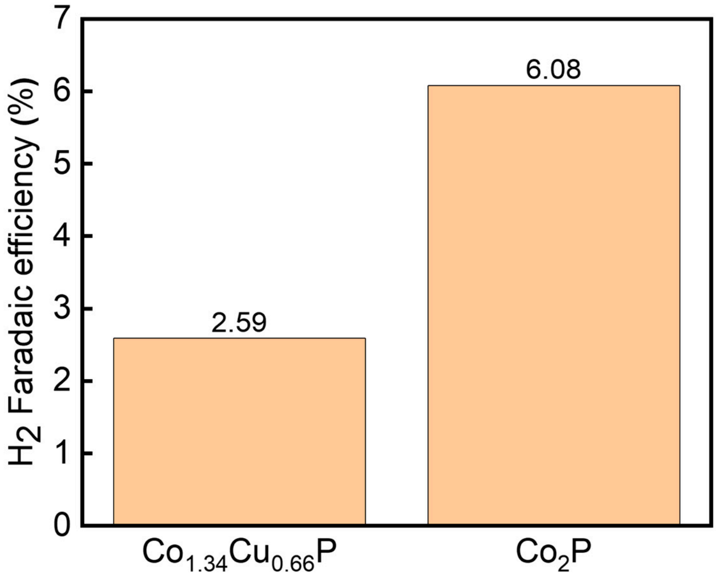

Figure. S8. Faradaic efficiency of H_2 evolution for np-$Co_{1.34}Cu_{0.66}P$ and Co_2P

Table S1 Mass of raw materials (Co, Co_2P, Cu) required to prepare 15 g of alloy with different Co:Cu:P atomic ratios.

Samples	Co (g)	Cu (g)	Co_2P (g)
$Co_{75}Cu_5P_{20}$ precursor alloy	5.79	0.89	8.31
$Co_{70}Cu_{10}P_{20}$ precursor alloy	4.95	1.81	8.24
$Co_{65}Cu_{15}P_{20}$ precursor alloy	4.10	2.73	8.17
$Co_{80}P_{20}$ precursor alloy	6.62	/	8.38

Table S2 Elemental composition of np-$Co_{2-x}Cu_xP$ measured by EDS.

Test Elements	Atomic percentage of np-$Co_{1.94}Cu_{0.06}P$	Atomic percentage of np-$Co_{1.34}Cu_{0.66}P$	Atomic percentage of np-$Co_{1.04}Cu_{0.96}P$
P	35.2 %	29.8 %	26.13 %
Cu	1.8 %	15.8 %	24.05 %
Co	63.0 %	54.4 %	49.82 %

Table S3 Elemental composition of Cu-Co-P samples measured by ICP.

Samples	Co (at %)	Cu (at %)	P (at %)
np-$Co_{1.94}Cu_{0.06}P$	66.0 %	1.6 %	32.4 %
np-$Co_{1.34}Cu_{0.66}P$	59.5 %	10.6 %	29.9 %
np-$Co_{1.04}Cu_{0.96}P$	54.3 %	19.0 %	26.7 %
np-Co_2P	66.9 %	/	33.1 %
$Co_{75}Cu_5P_{20}$ precursor alloy	75.8 %	5.2 %	19.1 %
$Co_{70}Cu_{10}P_{20}$ precursor alloy	71.8 %	10.5 %	17.7 %
$Co_{65}Cu_{15}P_{20}$ precursor alloy	67.7 %	14.5 %	17.8 %
$Co_{80}P_{20}$ precursor alloy	81.4 %	/	18.6 %

Table S4 Comparison table of Faradaic efficiency between np-$Co_{1.34}Cu_{0.66}P$ catalyst and reported catalysts.

Catalysts	NH_3 Faradaic efficiency (%)	Potential (V vs. RHE)	Ref.
np-$Co_{1.34}Cu_{0.66}P$	93.4	-0.3	This work
Cu/Cu_2O NWAs	81.2	-0.9	[1]
RuPdPtCu	79.8	-0.95	[2]
Co-CuOx	53.5	-1.13	[3]
Co/CoO NSAs	91.2	-1.1	[4]
Pd-NDs/Zr-MOF	58.1	-1.3	[5]
TiO_{2-x}	85	-1.3	[6]
Co-Fe/Fe_2O_3	85.2	-1.6	[7]
Cu-N-C SAC	84.7	-0.95	[8]
Fe_2Co-MOF	90.55	-1	[9]
Cu-Ni	88	-1.1	[10]
Cu@C	85.6	-0.9	[11]

References

[1] Y. Wang, W. Zhou, R. Jia, Y. Yu, B. Zhang, Unveiling the activity origin of a copper‐based electrocatalyst for selective nitrate reduction to ammonia, Angew Chem Int Edit 59 (2020) 5350-5354. https://doi.org/10.1002/anie.201915992

[2] M. Yang, B. Li, S. Li, Q. Dong, Z. Huang, S. Zheng, Y. Fang, G. Zhou, X. Chen, X. Zhu, T. Li, M. Chi, G. Wang, L. Hu, Z.J. Ren, Highly Selective Electrochemical Nitrate to Ammonia Conversion by Dispersed Ru in a Multielement Alloy Catalyst, Nano Lett 23 (2023) 7733-7742. https://doi.org/10.1021/acs.nanolett.3c01978.

[3] Y. Li, J. Ma, Z. Wu, Z. Wang, Direct Electron Transfer Coordinated by Oxygen Vacancies Boosts Selective Nitrate Reduction to N_2 on a Co-CuO_x Electroactive Filter, Environ. Sci. Technol. 56 (2022) 8673-8681. https://pubs.acs.org/doi/10.1021/acs.est.1c05841

[4] Y. Yu, C. Wang, Y. Yu, Y. Wang, B. Zhang, Promoting selective electroreduction of nitrates to ammonia over electron-deficient Co modulated by rectifying Schottky contacts, Sci China Chem 63 (2020) 1469-1476. https://doi.org/10.1007/s11426-020-9795-x

[5] M. Jiang, J. Su, X. Song, P. Zhang, M. Zhu, L. Qin, Z. Tie, J.-L. Zuo, Z. Jin, Interfacial Reduction Nucleation of Noble Metal Nanodots on Redox-Active Metal–Organic Frameworks for High-Efficiency Electrocatalytic Conversion of Nitrate to Ammonia, Nano Lett 22 (2022) 2529-2537. https://doi.org/10.1021/acs.nanolett.2c00446.

[6] R. Jia, Y. Wang, C. Wang, Y. Ling, Y. Yu, B. Zhang, Boosting Selective Nitrate Electroreduction to Ammonium by Constructing Oxygen Vacancies in TiO_2, ACS Catal 10 (2020) 3533-3540. https://doi.org/10.1021/acscatal.9b05260.

[7] S. Zhang, M. Li, J. Li, Q. Song, X. Liu, High-ammonia selective metal-organic framework-derived Co-doped Fe/Fe_2O_3 catalysts for electrochemical nitrate reduction, Proc Natl Acad Sci USA, 119 (2022) e2115504119.

https://doi.org/10.1073/pnas.2115504119.
[8] J. Yang, H. Qi, A. Li, X. Liu, X. Yang, S. Zhang, Q. Zhao, Q. Jiang, Y. Su, L. Zhang, Potential-driven restructuring of Cu single atoms to nanoparticles for boosting the electrochemical reduction of nitrate to ammonia, J. Am. Chem. Soc. 144 (2022) 12062-12071. https://pubs.acs.org/doi/10.1021/jacs.2c02262.
[9] Y. Lv, S.W. Ke, Y. Gu, B. Tian, L. Tang, P. Ran, Y. Zhao, J. Ma, J.L. Zuo, M. Ding, Highly Efficient Electrochemical Nitrate Reduction to Ammonia in Strong Acid Conditions with Fe_2M-Trinuclear-Cluster Metal-Organic Frameworks, Angew Chem Int Edit 62 (2023). https://doi.org/10.1002/anie.202305246.
[10] Y. Bu, C. Wang, W. Zhang, X. Yang, J. Ding, G. Gao, Electrical pulse-driven periodic self-repair of Cu-Ni tandem catalyst for efficient ammonia synthesis from nitrate, Angew Chem Int Edit 135 (2023) e202217337.
https://doi.org/10.1002/anie.202217337
[11] Z. Song, Y. Liu, Y. Zhong, Q. Guo, J. Zeng, Z. Geng, Efficient electroreduction of nitrate into ammonia at ultralow concentrations via an enrichment effect, Adv. Mater. 34 (2022) 2204306. https://doi.org/10.1002/adma.202204306.
